# Continuous β-Amyloid CSF/PET Imbalance Model to Capture Alzheimer Disease Heterogeneity

**DOI:** 10.1212/WNL.0000000000209419

**Published:** 2024-06-11

**Authors:** Sophie E. Mastenbroek, Arianna Sala, David Vállez García, Mahnaz Shekari, Gemma Salvadó, Luigi Lorenzini, Leonard Pieperhoff, Alle Meije Wink, Isadora Lopes Alves, Robin Wolz, Craig Ritchie, Mercè Boada, Pieter Jelle Visser, Marco Bucci, Gill Farrar, Oskar Hansson, Agneta K. Nordberg, Rik Ossenkoppele, Frederik Barkhof, Juan Domingo Gispert, Elena Rodriguez-Vieitez, Lyduine E. Collij, for the Alzheimer's Disease Neuroimaging Initiative on behalf of the AMYPAD consortium

**Affiliations:** From the Department of Radiology and Nuclear Medicine (S.E.M., D.V.G., L.L., L.P., A.M.W., F.B., L.E.C.), Vrije Universiteit Amsterdam, Amsterdam University Medical Center, location VUmc; Amsterdam Neuroscience (S.E.M., D.V.G., L.L., L.P., A.M.W., F.B., L.E.C.), Brain Imaging, the Netherlands; Clinical Memory Research Unit (S.E.M., G.S., O.H., R.O., L.E.C.), Department of Clinical Sciences Malmö, Lund University; Division of Clinical Geriatrics (A.S., M. Bucci, A.K.N., E.R.-V.), Center for Alzheimer Research, Department of Neurobiology, Care Sciences and Society, Karolinska Institutet, Stockholm, Sweden; Coma Science Group (A.S.), GIGA-Consciousness, University of Liège; Centre du Cerveau2 (A.S.), University Hospital of Liège, Belgium; Barcelonaβeta Brain Research Center (BBRC) (M.S., G.S., J.D.G.), Pasqual Maragall Foundation; IMIM (Hospital del Mar Medical Research Institute) (M.S., J.D.G.), Barcelona; Centro de Investigación Biomédica en Red de Fragilidad y Envejecimiento Saludable (M.S., J.D.G.), Instituto de Salud Carlos III, Madrid; Universitat Pompeu Fabra (M.S.), Barcelona, Spain; Brain Research Center (I.L.A.), Amsterdam, the Netherlands; IXICO (R.W.), London; Centre for Clinical Brain Sciences (C.R.), University of Edinburgh, United Kingdom; Ace Alzheimer Center Barcelona (M. Boada), Universitat Internacional de Catalunya, Spain; Networking Research Center on Neurodegenerative Diseases (CIBERNED) (M. Boada), Instituto de Salud Carlos III, Madrid, Spain; Alzheimer Center Amsterdam (P.J.V., R.O.), Neurology, Vrije Universiteit Amsterdam, Amsterdam UMC location VUmc; Amsterdam Neuroscience (P.J.V.), Neurodegeneration; Alzheimer Center Limburg (P.J.V.), School for Mental Health and Neuroscience, Maastricht University, the Netherlands; Division of Neurogeriatrics (P.J.V.), Department of Neurobiology, Care Sciences and Society, Karolinska Institutet, Stockholm, Sweden; Theme Inflammation and Aging (M. Bucci, A.K.N.), Karolinska University Hospital, Stockholm, Sweden; GE Healthcare (G.F.), Amersham, United Kingdom; Memory Clinic (O.H.), Skåne University Hospital, Malmö, Sweden; and Centre for Medical Image Computing (F.B.), and Queen Square Institute of Neurology, UCL, London, United Kingdom.

## Abstract

**Background and Objectives:**

Discordance between CSF and PET biomarkers of β-amyloid (Aβ) might reflect an imbalance between soluble and aggregated species, possibly reflecting disease heterogeneity. Previous studies generally used binary cutoffs to assess discrepancies in CSF/PET biomarkers, resulting in a loss of information on the extent of discordance. In this study, we (1) jointly modeled Aβ-CSF/PET data to derive a continuous measure of the imbalance between soluble and fibrillar pools of Aβ, (2) investigated factors contributing to this imbalance, and (3) examined associations with cognitive trajectories.

**Methods:**

Across 822 cognitively unimpaired (n = 261) and cognitively impaired (n = 561) Alzheimer's Disease Neuroimaging Initiative individuals (384 [46.7%] females, mean age 73.0 ± 7.4 years), we fitted baseline CSF-Aβ_42_ and global Aβ-PET to a hyperbolic regression model, deriving a participant-specific Aβ-aggregation score (standardized residuals); negative values represent more soluble relative to aggregated Aβ and positive values more aggregated relative to soluble Aβ. Using linear models, we investigated whether methodological factors, demographics, CSF biomarkers, and vascular burden contributed to Aβ-aggregation scores. With linear mixed models, we assessed whether Aβ-aggregation scores were predictive of cognitive functioning. Analyses were repeated in an early independent validation cohort of 383 Amyloid Imaging to Prevent Alzheimer's Disease Prognostic and Natural History Study individuals (224 [58.5%] females, mean age 65.2 ± 6.9 years).

**Results:**

The imbalance model could be fit (pseudo-*R*^2^ = 0.94) in both cohorts, across CSF kits and PET tracers. Although no associations were observed with the main methodological factors, lower Aβ-aggregation scores were associated with larger ventricular volume (β = 0.13, *p* < 0.001), male sex (β = −0.18, *p* = 0.019), and homozygous *APOE*-ε4 carriership (β = −0.56, *p* < 0.001), whereas higher scores were associated with increased uncorrected CSF p-tau (β = 0.17, *p* < 0.001) and t-tau (β = 0.16, *p* < 0.001), better baseline executive functioning (β = 0.12, *p* < 0.001), and slower global cognitive decline (β = 0.14, *p* = 0.006). In the validation cohort, we replicated the associations with *APOE*-ε4, CSF t-tau, and, although modestly, with cognition.

**Discussion:**

We propose a novel continuous model of Aβ CSF/PET biomarker imbalance, accurately describing heterogeneity in soluble vs aggregated Aβ pools in 2 independent cohorts across the full Aβ continuum. Aβ-aggregation scores were consistently associated with genetic and AD-associated CSF biomarkers, possibly reflecting disease heterogeneity beyond methodological influences.

## Introduction

The accumulation of β-amyloid (Aβ) plaques in the brain is considered one of the main pathologic hallmarks of Alzheimer disease (AD) and can be measured directly using PET-imaging, or indirectly as Aβ_42_ in the CSF.^1,2^ Although these measures are generally regarded as interchangeable biomarkers of Aβ-status, discordance occurs in 10%–20% of the AD continuum.^2-4^ It has been suggested that these biomarkers measure biochemically distinct Aβ pools, with CSF reflecting soluble Aβ and PET fibrillary aggregates.^5^ Hence, discrepancies might indicate an imbalance between different Aβ-species, possibly reflecting distinct disease pathways.^2^ To date, models characterizing discordance have been suboptimal, and consequently, its underlying factors and clinical value remain poorly understood.

Previous studies investigating discordance generally used binary cutoffs to assign participants into discordant groups of either isolated low CSF-Aβ_42_ (CSF+/PET−) or isolated elevated Aβ-PET retention (CSF−/PET+).^1-4,6-9^ Based on this approach, differences between CSF+/PET− and CSF−/PET+ groups in demographics, cognitive, clinical, genetic, and/or AD biomarker profiles and trajectories have been reported, with, however, discrepant findings among studies.^1-4,6-8^ These inconsistencies could be due to cohort-related differences,^10^ varying statistical power, or methodological factors,^10,11^ such as the use of different cutoffs to establish Aβ biomarker status.^12-14^ Other limitations of using discrete cutoff values include a loss of information on the extent of discordance and limiting analyses to early disease stages where discordance is most frequently observed.

Therefore, we propose a continuous model of soluble/aggregated Aβ-imbalance, by jointly modelling Aβ-CSF/PET data of cognitively unimpaired (CU) and cognitively impaired (CI) individuals and deriving a participant-specific Aβ-aggregation score. This approach provides us with a single measure capturing the full range of possible Aβ biomarker discordance. We investigated the association of Aβ-aggregation scores with (1) methodological factors, to support that the observed results are not due to methodology; (2) demographics, CSF biomarkers, and vascular burden, to assess biological factors that may promote imbalance between soluble and aggregated Aβ; and (3) baseline and longitudinal cognition, to investigate clinical relevance. Finally, we investigated the generalizability of the model to an independent, heterogeneous, multicohort validation data set across multiple CSF kits and PET tracers.

## Methods

### Study Cohorts

Data were obtained from the Alzheimer's Disease Neuroimaging Initiative (ADNI) database (adni.loni.usc.edu) and from the Amyloid Imaging to Prevent Alzheimer's Disease (AMYPAD) Prognostic and Natural History Study (PNHS) (version number v202306; doi: 10.5281/zenodo.8017084data; release January 20, 2023).^15^ The ADNI study was launched in 2003 as a public-private partnership, led by Principal Investigator Michael W. Weiner, MD. The primary goal of ADNI has been to test whether MRI, PET, other biological markers, and clinical and neuropsychological assessment can be combined to measure the progression of mild cognitive impairment (MCI) and early AD. The AMYPAD-PNHS is a pan-European cohort recruiting from 17 sites across 10 parent cohorts (PCs) and collects information on cognitive functioning, disease biomarkers, and traditional risk factors. Its main focus is on the earliest stages of AD, with inclusion criteria of Clinical Dementia Rating (CDR) ≤0.5 and age >50 years.

### Standard Protocol Approvals, Registrations, and Patient Consents

The study was approved by the Ethics Committees or Institutional Review Boards of each ADNI site before study initiation. All participants gave written informed consent (ClinicalTrials.gov registry numbers: ADNI GO: NCT01078636; ADNI 1: NCT00106899; ADNI 2: NCT01231971; AMYPAD-PNHS EudraCT: 2018-002277-22).

### Participants

The discovery sample included 822 ADNI participants of whom 261 were CU (healthy controls: n = 173, subjective memory complaints: n = 88) and 561 CI (MCI: n = 427, dementia: n = 134). All participants underwent CSF-Aβ_42_ sampling and [^18^F]florbetapir (FBP) PET imaging at baseline, within a time interval of 90 days (mean = 10.1, SD = 13.8 days; median = 6.0, interquartile range [IQR] = 1.0–13.0 days), obtained between April 2010 and April 2014 at 57 ADNI sites. Only those participants who had CSF-Aβ_42_ levels quantified through mass spectrometry, deemed as the gold standard in CSF protein quantification,^16^ were included.

The validation sample included 383 AMYPAD-PNHS participants of whom 326 were CU and 57 had MCI as defined by a CDR score of 0.5. These participants were selected from 3 PCs within the AMYPAD-PNHS, including the European Prevention of Alzheimer's Dementia Longitudinal Cohort Study (EPAD-LCS) (n = 202),^17^ the Alzheimer's and Family cohort (ALFA+) (n = 132),^18^ and the Fundació ACE Healthy Brain Initiative (FACEHBI) study (n = 49).^19^ All participants underwent CSF-Aβ_42_ sampling and [^18^F]florbetaben (FBB) or [^18^F]flutemetamol (FMM) PET imaging at baseline within 1 year of each other (mean = 124.0, SD = 94.60 days; median = 99.0, IQR = 43.5–187.5 days).

### CSF Measures

In the discovery cohort, ADNI lumbar CSF samples were acquired in the morning following overnight fasting, as described previously on the ADNI website (adni.loni.usc.edu/). CSF-Aβ_42_, Aβ_40_, and Aβ_38_ were quantified using 2D-UPLC-tandem mass spectrometry.^20^ CSF phosphorylated-tau (p-tau) and total-tau (t-tau) levels were derived through the multiplex xMAP Luminex platform (Luminex Corp, Austin, TX) with an INNO-BIA AlzBio3 immunoassay kit (Innogenetics, Ghent, Belgium).^21^

For the validation cohort, lumbar CSF samples were collected and analyzed separately in each PNHS PC according to local procedures.^18,22,23^ All participants had available CSF-Aβ_42_, p-tau, and t-tau measurements. For EPAD-LCS and ALFA+, samples were quantified using the Roche Cobas Elecsys System at the Clinical Neurochemistry Laboratory at the University of Gothenburg, Sweden.^24-26^ FACEHBI samples were analyzed using the ELISA immunoassay (INNOTEST Fujirebio Europe, Göteborg, Sweden).^22^ To pool data across PCs and kits, raw CSF data were standardized to *z*-scores based on cohort-specific CU reference groups (baseline visit; CDR = 0; Mini-Mental State Examination [MMSE] >27; *APOE*-ε4 noncarrier; ≤70 years of age; Aβ-negative [Aβ-PET Centiloids (CL) <12]).

### Aβ-PET Acquisition and Processing

Full details of PET procedures in the ADNI discovery cohort are described elsewhere.^27^ Briefly, FBP PET images were obtained 50–70 minutes postinjection (p.i.) using various PET scanners. Standardized uptake value ratio (SUVr) images were created using the whole cerebellum as the reference region. Following the standard ADNI pipeline, scans were processed using FreeSurfer version 7.1 to extract a weighted global SUVr measure across frontal, anterior/posterior cingulate, lateral parietal, and lateral temporal lobes. Finally, SUVrs were transformed to the CL scale^28^ to enable comparisons with the validation cohort.

In the validation cohort, FMM (EPAD-LCS [n = 126] and ALFA+) and FBB (EPAD-LCS [n = 76] and FACEHBI) PET scans were conducted according to the standard protocol for each tracer.^29,30^ Specifically, 4 frames of 5 minutes each were acquired 90–110 minutes p.i. of 185 megabecquerel (MBq) (±10%) for FMM and 350 MBq (±20%) for FBB, using a variety of PET scanners. Image analysis was performed centrally using IXICO's in-house fully automated workflow. PET frames were coregistered, averaged, and aligned to the corresponding MRI scan, which was parcellated using a participant-specific multiatlas approach, that is, the learning embeddings for atlas propagation (LEAP) parcellation procedure.^31^ Since Aβ-PET scans were obtained from a variety of scanners and sites, an image harmonization standard operational procedure has been developed in collaboration with the European Association of Nuclear Medicine initiative EARL (earl.eanm.org/) to harmonize quantification in nuclear medicine imaging.^32^ SUVr images were obtained using LEAP parcellation masks with the whole cerebellum reference region in native space. To pool Aβ-PET data across PCs, SUVrs were transformed to CLs using the standard GAAIN target region as a measure of global amyloid burden.^28,33^

### MRI Acquisition and Processing

At baseline, a subset of participants in the discovery cohort underwent MRI as described previously.^34^ From T1-weighted images, we derived ventricular volumes measured as the bilateral sum of the lateral ventricles; third, fourth, and fifth ventricles; and the choroid plexus (n = 652). In addition, white matter hyperintensity (WMH) volumes were assessed based on a Bayesian approach to segment high-resolution 3D T1-weighted and fluid-attenuated inversion recovery (FLAIR) sequences (n = 776).^35^ All MRI measures were corrected for total intracranial volume.

Within 1 year of baseline, a subset of participants in the validation cohort underwent MRI according to PC-specific protocols (n = 336). MRI images were acquired on a Siemens Healthineers, Philips Healthcare, or GE Healthcare scanner (EPAD-LCS)^36^; a Philips Ingenia CX 3T scanner (ALFA+)^37^; and a 1.5-T Siemens Magneton Aera scanner (FACEHBI).^38^ T1-weighted scans were processed centrally, including motion correction, removal of nonbrain tissue, and parcellation into FreeSurfer v7.1.1-based regions of interest. Parcellations were visually quality controlled. Ventricular volumes were computed identically to ADNI. Since WMH volumes were not available in the validation cohort, Fazekas visual read in the deep white matter was assessed from FLAIR sequences by local readers for 228 participants.

### Cognitive Assessments

All participants in the discovery cohort underwent a complete cognitive assessment within 1 year of baseline (mean time interval ranged from 0.4–9.4 months across cognitive domains), and a subset was followed over time (n = 792), with mean follow-up time ranging from 3.6 to 3.8 years across domains. Cognitive measures used in this study included global cognition assessed with the MMSE and Alzheimer's Disease Assessment Scale-cognitive subscale (ADAS-Cog11), a composite score of memory (ADNI-MEM) performance, and a composite score of executive functioning (ADNI-EF) performance. As described more in-depth previously,^39,40^ composite scores were created by applying latent modelling to a range of cognitive tests (ADNI-MEM: memory items from the MMSE and ADAS-Cog11, immediate and delayed recall and recognition scores from the Rey Auditory Verbal Learning Test, and immediate and delayed recall scores from Logical Memory I of the Wechsler Memory Scale Revised; ADNI-EF: the Wechsler Adult Intelligence Scale Revised Digit Symbol Substitution Test, the Digit span Backward test, Trail Making Test A and B, animals and vegetables category fluency tests, and the clock drawing test).

For participants in the validation cohort, we selected similar global, memory, and executive functioning tests within 1 year of baseline (mean time interval ranged from 1.8 to 4.0 months across tests) and over time, with mean follow-up time ranging from 2.7 to 3.6 years between tests. Tests included the MMSE, immediate and delayed recall and recognition tests (EPAD-LCS: subdomains of the Repeatable Battery for the Assessment of Neuropsychological status; ALFA+: the Free and Cued Selective Reminding test; FACEHBI: Wechsler Memory Scale III), a categorical (animals or vegetables) fluency test, the Digit Span Backward test, and Trail Making Test B corrected for A. Sample sizes varied by test, with longitudinal analyses including all participants with at least 1 measurement. For each cognitive test, cohort-specific *z*-scores were calculated based on the aforementioned CU reference group.

### Continuous Aβ CSF/PET Imbalance Modelling

**Figure 1:**
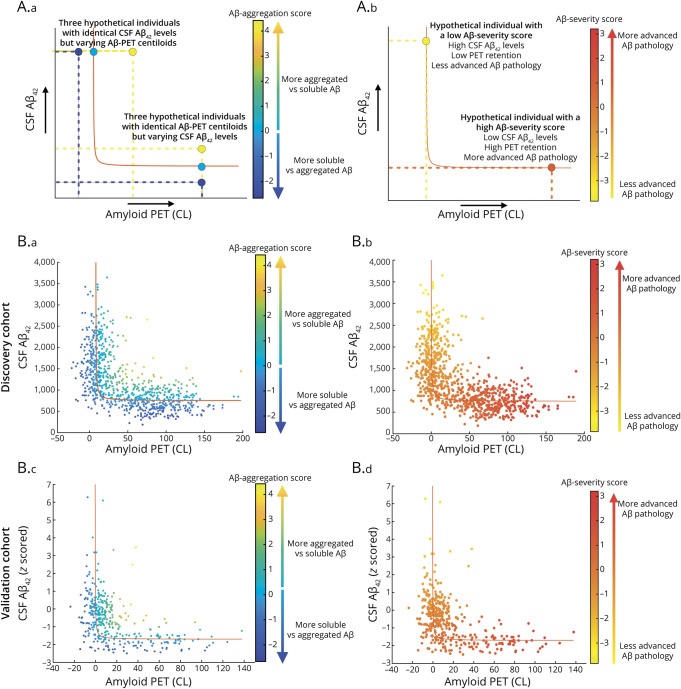
Continuous Aβ CSF/PET Imbalance Model Applied to the Discovery and Validation Cohort (A) Illustration of the interpretation of Aβ-aggregation (left) and Aβ-severity (right) measures. Aβ-aggregation scores reflect the relative imbalance between CSF and PET biomarkers of Aβ, with a value of 0 (light blue) representing no imbalance, positive values (green-yellow) representing more aggregated relative to soluble Aβ, and negative values (dark blue) representing more soluble relative to aggregated Aβ. The top left part of the figure shows 3 hypothetical datapoints with identical CSF Aβ_42_ levels on the y-axis, but varying levels of Aβ-PET CL on the x-axis and the effect on the Aβ-aggregation measure. Aβ-severity scores indicate where along the hyperbolic model an individual is located, with negative values (orange-yellow) representing an individual at the beginning of the regression line/Aβ aggregation trajectory with less advanced Aβ pathology, and positive values (red) representing an individual at the end of the regression line/Aβ aggregation trajectory with more advanced Aβ pathology. (B) Scatterplots of the hyperbolic relationship between CSF-Aβ_42_ and Aβ-PET CLs in the discovery cohort (top row) and validation cohort (bottom row), color-coded for Aβ-aggregation scores (left) and Aβ-severity (right). Aβ = β-amyloid; CL = Centiloid.

Modelling was performed separately for the discovery (CU and CI combined) and validation cohort, using in-house code written in MATLAB (version R2022a; The Mathworks, Matwick, MA). More detailed information on the statistical modelling is described in the Supplementary Methods. Briefly, we iteratively fitted a hyperbolic regression model between baseline CSF-Aβ_42_ (pg/mL in the main cohort, *z*-scored in the validation cohort) and Aβ-PET CL data, by minimizing the sum of the Euclidean distance of the experimental points to the fitted line (Figure 1B).

Next, from this single model, we derived 2 subject-specific measures of (1) the relative imbalance between soluble (CSF) and aggregated (PET) Aβ, termed Aβ-aggregation, and (2) the extent of Aβ-pathology, termed Aβ-severity (Figure 1A). Aβ-aggregation scores were calculated as the difference between the observed and predicted data point (i.e., the standardized Euclidean distance of the observed data point to the fitted line). A positive score denotes a higher PET CL value than expected for a given value of CSF-Aβ_42_ (i.e., more aggregated relative to soluble Aβ), whereas a negative score denotes lower CSF-Aβ_42_ values than expected for a given value of PET CL (i.e., more soluble relative to aggregated Aβ). In turn, Aβ-severity indicates where—along the hyperbolic regression line—the datapoint is located (i.e., the standardized Euclidean distance between the individual predicted value and the median of all predicted values). A positive score reflects more advanced Aβ-pathology and a negative score reflects less advanced Aβ-pathology. Figure 1A shows several hypothetical data points and their respective Aβ-aggregation and Aβ-severity scores.

### Statistical Analysis

Differences in sample characteristics between diagnostic groups and between cohorts were assessed using 2-sample *t* tests for continuous variables, with Wilcoxon tests in case of nonnormality, and chi-square tests for categorical variables. For subsequent statistical analyses, we included participants with ventricular volume measures (n_discovery_ = 652, n_validation_ = 336), with varying sample sizes across statistical models.

In the discovery cohort, we performed separate linear regression models predicting the Aβ-aggregation score with (1) methodological factors (Δt _CSF/PET-measures_ and ventricular volume), (2) demographics (age, sex, years of education, and number of *APOE*-ε4 copies), (3) CSF biomarkers (Aβ_38_ and Aβ_40_, p-tau, and t-tau), and (4) vascular burden (WMH volumes). CSF predictors were tested as raw data and divided by CSF-Aβ_40_. For methodological and demographic factors, predictors were tested concurrently, while CSF and vascular predictors were tested individually and corrected for age, sex, and number of *APOE*-ε4 copies. All models were adjusted for Aβ-severity as a measure of total Aβ-pathology burden and for ventricular volume. To determine whether Aβ-aggregation scores predicted baseline (Aβ-aggregation at time = 0) and longitudinal (Aβ-aggregation × time) cognition, we ran domain-specific linear mixed models with participant-specific random intercept and slope. Covariates were Aβ-severity, ventricular volume, age, sex, years of education, and number of *APOE*-ε4 copies. We assessed whether the associations with imbalance were dependent on the extent of Aβ-pathology by adding a 2-way interaction term to the models at baseline and a 3-way interaction for longitudinal cognition. As sensitivity analyses, all above-stated analyses were stratified for cognitive status (CU and CI) and corrected for CSF dynamics by computing CSF-Aβ_42/40_ and Aβ-PET CL-derived Aβ-aggregation and Aβ-severity scores.

Similar models were performed in the validation cohort, where CSF kit and PET tracer were added as methodological factors, Fazekas visual read was used instead of WMH volume, and cognitive functioning was assessed in specific tests as described under cognitive assessments. Sensitivity analyses were not conducted because of a lack of data on CSF Aβ_40_ and too few CI individuals for stratification.

Statistical analyses were performed in R version 4.2.0.^41^ Significance was set at 2-sided *p* < 0.05. *p* Values regarding models with CSF biomarkers and cognition were false discovery rate–corrected for multiple comparisons based on the number of models. Regression coefficients are reported as standardized betas (βs).

### Data Availability

The data that support the findings of this study are openly available on request on the ADNI open-source database and through the Alzheimer's Disease Data Initiative website for AMYPAD-PNHS (alzheimersdata.org/). An in-house Matlab code to derive Aβ-aggregation and Aβ-severity scores is available on the AMYPAD website (amypad.eu/resources/software/).

## Results

### Participant Characteristics

Baseline demographics of the discovery and validation cohorts are summarized in Table 1. Compared with the discovery cohort, the validation cohort was younger (73.0 ± 7.4 vs 65.2 ± 6.9 years, *t* = 17.4, *p* < 0.001), had a higher proportion of women (46.7% vs 58.5%, χ^2^ = 13.9, *p* < 0.001), fewer years of education (16.3 ± 2.6 vs 14.6 ± 3.8, W = 200,094, *p* < 0.001), a higher MMSE (27.6 ± 2.6 and 28.9 ± 1.5, W = 106,328, *p* < 0.001), smaller ventricles (0.03 ± 0.00 vs 0.02 ± 0.01, *t* = 7.3, *p* < 0.001), and lower amyloid burden (40.3 ± 44.1 vs 17.4 ± 27.8, W = 200,876, *p* < 0.001), illustrating the early nature of this cohort.

**Table 1:** Baseline Characteristics

	Discovery cohort				Validation cohort	
	All	CU	CI	*p* Value^a^	All	*p* Value^b^
N	822	261	561		383	
Age	73.0 (7.4)	73.8 (6.4)	72.6 (7.8)	0.025^e^	65.2 (6.9)	<0.001^e^
Female, n (%)	384 (46.7)	141 (54.0)	243 (43.3)	0.005^e^	224 (58.5)	<0.001^e^
Years of education	16.3 (2.6)	16.0 (2.7)	16.4 (2.6)	0.133	14.6 (3.8)	<0.001^e^
MMSE score	27.6 (2.6)	29.1 (1.1)	26.8 (2.8)	<0.001^e^	28.9 (1.5)	<0.001^e^
Missing, n (%)	0 (0.0)	0 (0.0)	0 (0.0)	—	17 (4.4)	—
*APOE*-ε4 copies, n (%)				<0.001^e^		0.132
0	451 (54.9)	189 (72.4)	262 (46.7)	—	189 (49.3)	—
1	293 (35.6)	66 (25.3)	227 (40.5)	—	159 (41.5)	—
2	78 (9.5)	6 (2.3)	72 (12.8)	—	33 (8.6)	—
Missing, n (%)	0 (0.0)	0 (0.0)	0 (0.0)	—	2 (0.5)	—
Ventricular volume^c^	0.03 (0.0)	0.02 (0.01)	0.03 (0.01)	0.001^e^	0.02 (0.01)	<0.001^e^
Missing, n (%)	170 (20.7)	62 (23.8)	108 (19.3)		47 (12.3)	—
Interval CSF and PET (d)	10.1 (13.8)	10.0 (14.4)	10.1 (13.6)	0.519	124.0 (94.6)	<0.001^e^
CSF-Aβ_42_^d^	1,200 (631)	1,460 (646)	1,080 (587)	<0.001^e^	−0.6 (1.3)	—
Global amyloid burden (CL)	40.3 (44.1)	22.2 (35.2)	48.7 (45.4)	<0.001^e^	17.4 (27.8)	<0.001^e^
Aβ-aggregation score	0 (1)	0.2 (1.0)	−0.1 (1.0)	0.001^e^	0 (1)	—
Aβ-severity score	0 (1)	−0.5 (0.9)	0.2 (1.0)	<0.001^e^	0 (1)	—

Abbreviations: Aβ = β-amyloid; CI = cognitively impaired; CL = Centiloid; CU = cognitively unimpaired; MMSE = Mini-Mental State Examination.

Data are reported as mean (SD), unless indicated otherwise.

a *p* Value indicates differences between CU and CI individuals in the discovery cohort.

b *p* Value indicates differences between the full discovery and validation cohorts.

c Ventricular volume is reported as the ratio between total ventricular volume (mm^3^) and intracranial volume (mm^3^).

d Raw data in pg/mL are shown for the discovery cohort, while *z*-scores are displayed for the validation cohort.

e *p* < 0.05.

### Continuous Imbalance Model

Figure 1B shows the fitted models for the discovery (a = 3,673.1, b = −2,924.9, c = −0.3; pseudo-*R*^2^ = 0.94) and validation cohort (a = 0.5, b = −2.2, c = −0.4; pseudo-*R*^2^ = 0.94), color coded by Aβ-aggregation (left) and Aβ-severity (right) scores. In both cohorts, a wide range of Aβ-aggregation values are observed across Aβ-severity (eFigure 1), indicating that imbalance permeates the entire Aβ accumulation process, with a similar Aβ-aggregation range across cohorts (eFigure 2). In the discovery cohort, Aβ-aggregation scores differed significantly (*p* = 0.001) between CU and CI groups, with an average of 0.2 ± 1.0 in CU participants and −0.1 ± 1.0 in CI participants (Table 1, eFigure 2A). As expected, CU individuals had on average a lower Aβ-severity score in comparison with CI (CU: −0.5 ± 0.9, CI: 0.2 ± 1.0; *p* < 0.001) (Table 1, eFigures 2C and 3).

### Methodologic and Biological Factors Promoting Imbalance

**Figure 2:**
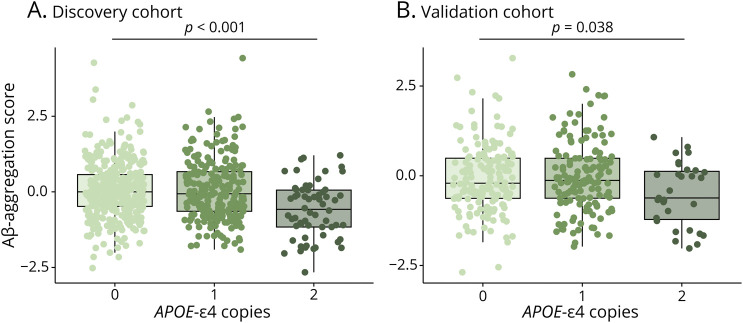
Association Between Aβ-Aggregation Score and Number of *APOE*-ε4 Copies Relationship between Aβ-aggregation scores and number of *APOE* ε4 copies in the (A) discovery cohort and (B) validation cohort. Aβ-aggregation indicates the imbalance between CSF and PET biomarkers of Aβ, with positive values representing more aggregated relative to soluble Aβ and negative values representing more soluble relative to aggregated Aβ. Unadjusted *p* values are shown. Aβ = β-amyloid.

In the discovery cohort, larger ventricular volume was associated with lower Aβ-aggregation scores (β = −0.13, *p* < 0.001; eFigure 4) (Table 2). Therefore, all subsequent analyses were adjusted for ventricular volume. Male sex (β = −0.18, *p* = 0.019) (eFigure 4) and carrying 2 *APOE*-ε4 copies (β = −0.56, *p* < 0.001, Figure 2A) were associated with lower Aβ-aggregation scores. None of the above-stated predictors showed an interaction with Aβ-severity. Increased concentrations of CSF biomarkers reflecting tau burden (p-tau: β = 0.17, *p* < 0.001), neurodegeneration (t-tau: β = 0.16, *p* < 0.001), and Aβ production (Aβ_38_: β = 0.48, *p* < 0.001; Aβ_40_: β = 0.51, *p* < 0.001) were related to higher Aβ-aggregation scores (eFigures 5 and 6). The relationships with Aβ_38_ (Aβ_38_ × Aβ-severity: β = 0.22, *p* < 0.001) and Aβ_40_ (Aβ_40_ × Aβ-severity: β = 0.56, *p* < 0.001) were exacerbated at more advanced levels of Aβ pathology. When correcting CSF biomarkers for Aβ_40_, an inversion of directionality of the associations was observed for p-tau (β = −0.19, *p* < 0.001) and t-tau (β = −0.19, *p* < 0.001). Associations remained significant after adjustment for multiple comparisons. Vascular burden was not significantly associated with the Aβ-aggregation measure and did not show a significant interaction with Aβ-severity.

**Table 2:** Baseline Associations Between Aβ-Aggregation Score and Methodological, Demographical, CSF Biomarker, and Vascular Variables

Predictor	Discovery cohort				Validation cohort			
	β (SE)	*p* Value	Adjusted *p* value	*R* ^2^	β (SE)	*p* Value	Adjusted *p* value	*R* ^2^
Methodological factors	n = 652				n = 336			
Interval CSF and PET	0.01 (0.04)	0.842	—	0.02	0.07 (0.06)	0.234	—	0.04
Ventricular volume	−0.13 (0.04)	<0.001^a^	—		−0.09 (0.05)	0.094	—	
CSF kit	—	—	—		−0.11 (0.21)	0.608	—	
PET tracer	—	—	—		0.09 (0.14)	0.520	—	
Demographics	n = 652				n = 334			
Age	0.04 (0.04)	0.384	—	0.07	0.02 (0.06)	0.752	—	0.06
Sex (ref male)	0.18 (0.08)	0.019^a^	—		0.09 (0.11)	0.402	—	
Years of education	−0.03 (0.04)	0.417	—		−0.07 (0.05)	0.189	—	
*APOE*-ε4 copies (ref 0)								
1	0.02 (0.09)	0.818	—		0.12 (0.12)	0.304	—	
2	−0.56 (0.14)	<0.001^a^	—		−0.41 (0.19)	0.038^a^	—	
CSF biomarkers	n = 652				n = 285			
Aβ production								
CSF-Aβ_38_	0.48 (0.04)	<0.001^a^	<0.001^a^	0.26	—	—	—	—
CSF-Aβ_40_	0.51 (0.04)	<0.001^a^	<0.001^a^	0.29	—	—	—	—
Tau burden								
CSF p-tau	0.17 (0.04)	<0.001^a^	<0.001^a^	0.09	0.11 (0.06)	0.070	0.070	0.07
Neurodegeneration								
CSF t-tau	0.16 (0.04)	<0.001^a^	<0.001^a^	0.09	0.14 (0.06)	0.020^a^	0.039^a^	0.08
Vascular burden	n = 612				n = 239			
WMH volumes	0.02 (0.04)	0.597	—	0.08	—	—	—	—
Fazekas score (ref 0)								
Score 1	—	—	—	—	−0.12 (0.14)	0.393	—	0.09
Score 2 or 3	—	—	—	—	−0.36 (0.22)	0.099	—	

Abbreviations: Aβ = β-amyloid; p-tau = phosphorylated tau; t-tau = total tau; WMH = white matter hyperintensity.

Results from linear models between Aβ-aggregation score as outcome and methodological factors, demographics, CSF biomarkers, and vascular burden as predictors. Betas are standardized, and the standard error is shown in brackets.

a *p* < 0.05. For CSF biomarkers, *p* values are shown unadjusted and adjusted for multiple comparisons.

Similarly to the discovery cohort, in the validation cohort, lower Aβ-aggregation scores were associated with homozygous *APOE*-ε4 carriership (β = −0.41, *p* = 0.038, Figure 2B) and higher scores with higher CSF t-tau levels (β = 0.14, *p* = 0.020, eFigure 5D) (Table 2). Associations remained significant after correction for multiple comparisons. CSF biomarkers showed a significant interaction with Aβ-severity, with higher concentrations being associated with a higher Aβ-aggregation score at more advanced levels of Aβ pathology (p-tau × Aβ-severity: β = 0.11, *p* = 0.024; t-tau × Aβ-severity: β = 0.19, *p* = 0.020). Ventricular volume (eFigure 4B), sex (eFigure 4D), CSF p-tau (eFigure 5B), vascular burden, or any of the additionally tested methodological factors was not associated with Aβ-aggregation.

### Associations With Baseline and Longitudinal Cognition

**Figure 3:**
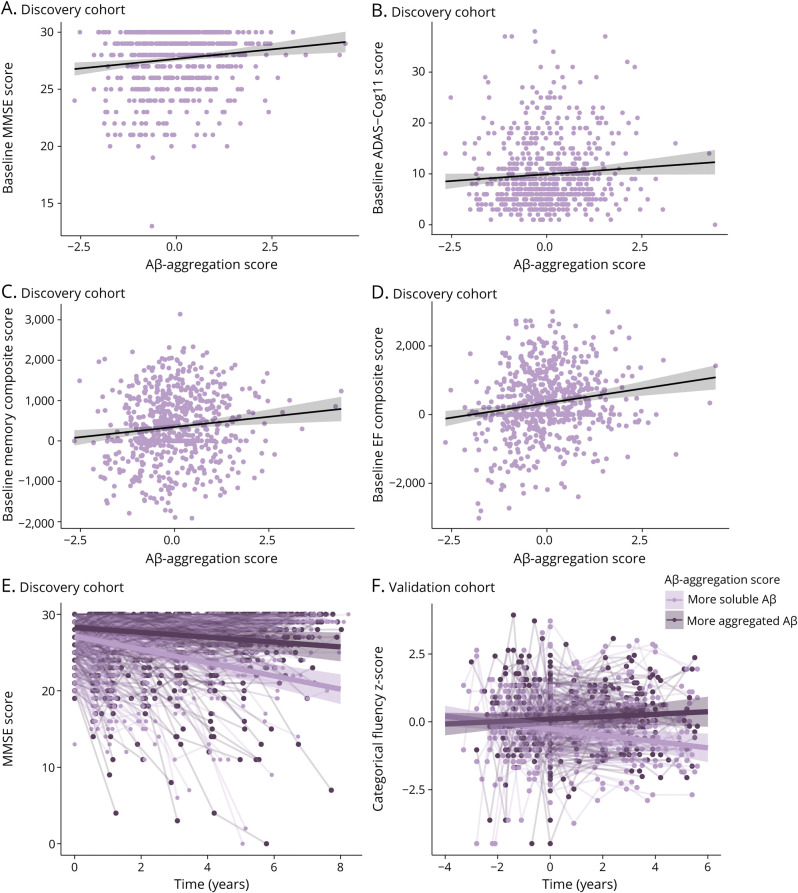
Predictive Value of Aβ-Aggregation Score on Baseline and Longitudinal Cognition (A–D) In the discovery cohort, scatterplots of the relationship are shown between Aβ-aggregation score and baseline cognitive performance on (A) the MMSE, with higher values representing better cognition; (B) the ADAS-Cog11, with higher values representing worse performance; (C) a memory composite with higher values representing better performance; and (D) an executive functioning composite with higher values representing better performance. (E, F) Association between Aβ-aggregation scores and longitudinal cognition with regression lines depicting a representative participant with a score of −2 (light purple) and 2 (dark purple). In the background, participant-specific trajectories are shown. Time was coded as years from baseline (baseline = 0). (E) In the discovery cohort, global cognition is shown as MMSE scores, (F) whereas in the validation cohort, semantic memory is shown as categorical fluency *z*-scores, with higher scores representing better performance. In the validation cohort, parent cohort specific *z*-scores were calculated for harmonization, based on a cognitively unimpaired reference sample. Aβ-aggregation indicates the imbalance between CSF and PET biomarkers of Aβ, with positive values representing more aggregated relative to soluble Aβ and negative values representing more soluble relative to aggregated Aβ. Aβ = β-amyloid; ADAS-Cog = Alzheimer's Disease Assessment Scale-Cognitive Subscale; EF = executive functioning; MMSE = Mini-Mental State Examination.

In the discovery cohort, higher Aβ-aggregation scores were related to better baseline global cognition measured with the MMSE (β = 0.23, *p* = 0.017), memory (β = 0.08, *p* = 0.013), and executive functioning (β = 0.14, *p* < 0.001) (Table 3, Figure 3, A, C, and D). By contrast, higher Aβ-aggregation scores were related to higher baseline ADAS-Cog11 scores, indicating worse global cognition (β = 0.09, *p* = 0.040, Figure 3B). Effects on baseline MMSE were exacerbated at more advanced Aβ levels (Aβ-imbalance × Aβ-severity: β = 0.18, *p* = 0.045). In addition, higher Aβ-aggregation scores were predictive of slower global cognitive decline over time as measured by the MMSE (β = 0.13, *p* = 0.007, Figure 3E), but not specific cognitive domains or in interaction with Aβ-severity. All associations remained after correction for multiple comparisons.

**Table 3:** Discovery Cohort: Aβ-Aggregation Score Predicting Baseline and Longitudinal Cognition

Outcome	Discovery cohort (n = 652)			
	β (SE)	*p* Value	Adjusted *p* value	*R* ^2^
Cross-sectional				
MMSE	0.23 (0.10)	0.017^a^	0.023^a^	0.20
ADAS-Cog11	0.56 (0.27)	0.042^a^	0.042^a^	0.05
ADNI-MEM	0.08 (0.03)	0.013^a^	0.023^a^	0.27
ADNI-EF	0.14 (0.04)	<0.001^a^	<0.001^a^	0.25
Longitudinal				
MMSE	0.14 (0.05)	0.006^a^	0.024^a^	0.09
ADAS-Cog11	0.10 (0.10)	0.308	0.308	0.08
ADNI-MEM	0.01 (0.01)	0.155	0.267	0.25
ADNI-EF	0.01 (0.01)	0.200	0.267	0.21

Abbreviations: Aβ = β-amyloid; ADAS-Cog = Alzheimer's Disease Assessment Scale-Cognitive Subscale; ADNI-EF = Alzheimer's Disease Neuroimaging Initiative executive functioning; ADNI-MEM = Alzheimer's Disease Neuroimaging Initiative memory; MMSE = Mini-Mental State Examination.

In the discovery cohort, results from separate linear mixed models between several cognitive domains as outcome and Aβ-aggregation score as predictor are shown at baseline and longitudinally. Betas are standardized, and standard errors are shown in brackets.

a *p* < 0.05. *p* Values are shown unadjusted and adjusted for multiple comparisons.

In the validation cohort, baseline cognition was not related to Aβ-aggregation or Aβ-aggregation × Aβ-severity. Higher Aβ-aggregation scores were associated with attenuated semantic memory decline over time (β = 0.04, *p* = 0.034; Table 4; Figure 3F). At high levels of Aβ pathology, high Aβ-aggregation scores were associated with slower memory decline (Aβ-aggregation × Aβ-severity × time: β = 0.04, *p* = 0.033). After adjustment for multiple comparisons, associations were no longer statistically significant.

**Table 4:** Validation Cohort: Aβ-Aggregation Score Predicting Baseline and Longitudinal Cognition

Outcome	Validation cohort				*R* ^2^
	n	β (SE)	*p* Value	Adjusted *p* value	
Cross-sectional					
MMSE	334	−0.05 (0.07)	0.495	0.693	0.15
Immediate recall	333	−0.01 (0.06)	0.908	0.951	0.26
Delayed recall	334	−0.04 (0.07)	0.951	0.951	0.29
Memory recognition	206	−0.20 (0.14)	0.167	0.390	0.16
Categorical fluency	334	0.09 (0.05)	0.074	0.390	0.17
Digit span backwards	177	−0.05 (0.06)	0.379	0.663	0.16
Trail Making Test B	176	−0.11 (0.07)	0.123	0.390	0.14
Longitudinal					
MMSE	334	−0.02 (0.02)	0.470	0.658	0.12
Immediate recall	333	0.03 (0.02)	0.103	0.240	0.45
Delayed recall	334	0.00 (0.02)	0.880	0.937	0.20
Memory recognition	206	0.07 (0.04)	0.067	0.235	0.12
Categorical fluency	334	0.04 (0.02)	0.034^a^	0.235	0.20
Digit span backwards	177	0.02 (0.02)	0.181	0.317	0.24
Trail Making Test B	176	0.00 (0.03)	0.937	0.937	0.14

Abbreviations: Aβ = β-amyloid; MMSE = Mini-Mental State Examination.

In the validation cohort, results from separate linear mixed models between several cognitive tests as outcome and Aβ-aggregation score as a predictor are shown at baseline and longitudinally. Betas are standardized, and standard errors are shown in brackets.

a *p* < 0.05. *p* Values are shown unadjusted and adjusted for multiple comparisons.

### Sensitivity Analyses in the Discovery Cohort

Associations with ventricular volume, CSF t-tau, CSF-Aβ_38_, and CSF-Aβ_40_ remained significant after stratification for baseline cognitive status (eTable 1). In both CU and CI groups, CSF Aβ_38_ and Aβ_40_ interacted with Aβ-severity so that higher CSF concentrations were associated with higher Aβ-aggregation scores at high levels of pathology (CU: Aβ_38_ × Aβ-severity β = 0.26, *p* < 0.001 and Aβ_40_ × Aβ-severity β = 0.28, *p* < 0.001; CI: Aβ_38_ × Aβ-severity β = 0.20, *p* < 0.001 and Aβ_40_ × Aβ-severity β = 0.24, *p* < 0.001). In CU individuals, a similar interaction was observed for CSF t-tau (β = −0.16, *p* = 0.015). Lower Aβ-aggregation scores for men were observed in CU individuals only (β = −0.36, *p* = 0.018), whereas lower scores in homozygous *APOE*-ε4 carriers (β = −0.62, *p* < 0.001) and higher scores with increased CSF p-tau levels (β = 0.20, *p* < 0.001) were observed in the CI group only. Although no further interactions with Aβ-severity were observed in the CI group, both education (β = 0.24, *p* = 0.002) and WMH volume (β = −0.25, *p* = 0.008) significantly interacted with Aβ-severity in CU individuals with—at high levels of pathology—more years of education being related to higher Aβ-aggregation scores and higher WMH volumes to lower Aβ-aggregation scores. Regarding cognition, baseline results were driven by the CI group and longitudinal findings or interactions could not be replicated in the CU or CI groups separately (eTable 2).

All above-mentioned analyses were repeated with Aβ-aggregation scores derived from CSF-Aβ_42/40_ (eTables 3 and 4). In line with the above-tested CSF biomarker analyses adjusted for Aβ_40_, the Aβ_42/40_-derived aggregation score was related to ventricular volume and CSF biomarkers in the opposite direction as compared with Aβ_42_-derived scores. This inversion of directionality was also observed in CI individuals, whereas no significant associations were found in the CU group. Similar results to the Aβ_42_-derived aggregation measure were found regarding demographics and cognition. More specifically, Aβ_42/40_-derived scores were negatively associated with carrying 2 *APOE*-ε4 copies in the whole cohort and CI individuals, and with male sex in CU individuals. In addition, higher Aβ_42/40_-derived aggregation scores were associated with slower longitudinal decline on a memory composite in CI individuals. However, this relationship did not survive correction for multiple comparisons, and associations with baseline cognition were not observed.

Results in the discovery and validation cohort without adjusting for ventricular volume are presented in eTables 5 and 6.

## Discussion

In this study, we propose a continuous model of Aβ CSF/PET biomarker imbalance, accurately describing heterogeneity in soluble vs aggregated Aβ pools in 2 independent cohorts. We derived participant-specific Aβ-aggregation scores, with negative values representing more soluble relative to aggregated Aβ and positive values reflecting more aggregated relative to soluble Aβ. We found that a lower Aβ-aggregation score was consistently, albeit weakly, associated with carrying 2 *APOE*-ε4 copies and lower concentrations of CSF t-tau in 2 independent cohorts, while no consistent association with methodological factors was reported. In the more pathologically advanced discovery cohort, a lower Aβ-aggregation score was additionally moderately related to worse cognition at baseline and follow-up. Altogether, our findings suggest that soluble/aggregated Aβ imbalance reflects disease heterogeneity beyond methodological influences.

Accumulation of fibrillar Aβ has been shown to be dependent on the amount of available Aβ and can be modified by the *APOE* genotype.^42^ *APOE*-ε4 seems to impair the balance between soluble and aggregated Aβ forms. In particular, *APOE*-ε4 was found to promote the accumulation of amorphous Aβ assemblies which might not be detected with PET imaging so that the same amount of soluble Aβ_42_ might result in a lower amount of fibrillar Aβ plaques in *APOE*-ε4 carriers. In line, we observed in both cohorts that homozygous *APOE*-ε4 carriership was related to an Aβ-imbalance toward more soluble CSF-Aβ_42_ relative to fibrillary PET retention. Of note, although the current work focused on global PET burden, a previous study suggested that the modulating effect of *APOE*-ε4 might be regionally dependent.^43^ It is therefore of future interest to investigate factors underlying regional imbalance patterns. In addition, we report a sex effect in the discovery cohort, with CU women having relatively more aggregated than soluble Aβ as compared with men. Underlying mechanisms remain to be further investigated, but sex hormones have been proposed to potentially play a role.^43^ Of interest, previous studies have emphasized the complexity of the interplay between different factors influencing Aβ fibrillization by showing interaction effects of sex and *APOE*-ε4 carriership on the extent of Aβ deposition.^44,45^

We observed in both the discovery and validation cohort that an imbalance toward more aggregated relative to soluble Aβ was associated with higher CSF t-tau concentrations. Similar associations were observed for CSF-Aβ_38_ and Aβ_40_ (note that the relationship could not be tested in the validation cohort due to lack of data), and CSF p-tau in the discovery cohort only. When we corrected for the possible influence of CSF dynamics in the ADNI cohort by adjusting CSF predictors for Aβ_40_ or by deriving Aβ-aggregation scores from CSF-Aβ_42/40_, we observed inversed directionality of associations with CSF biomarker concentrations, whereas associations with other variables remained in similar directions as with CSF-Aβ_42_–derived aggregation scores. A possible explanation is that Aβ biomarker imbalance might be greatly influenced by CSF dynamics, as illustrated by studies showing the importance of correcting for interindividual variability,^46^ and that using CSF-Aβ_42/40_ yields a lower frequency of discordance with Aβ-PET as compared with CSF-Aβ_42_.^42,47,48^ Therefore, it could be that individuals with relatively low CSF-Aβ_42_ concentrations generally have low CSF protein levels due to specific factors affecting general production and clearance dynamics, such as enlarged ventricles or inherent lower protein production.

Finally, we observed a modest association between cognition and Aβ-imbalance in the discovery cohort. More soluble relative to aggregated Aβ was associated with worse baseline global, memory, and executive functioning performance and with steeper global cognitive decline. All models were adjusted for Aβ-severity, indicating that associations with cognition persist even when correcting for variations in Aβ accumulation progression. A possible explanation is that individuals with relatively more soluble Aβ might have more toxic/detrimental oligomeric Aβ-species.^49^ These prefibrillary oligomers are Aβ-assemblies that exist along the aggregation pathway from soluble Aβ-peptides to fibrillar plaques and are not (yet) detectable with PET. Hence, an increase in oligomeric species would explain reduced soluble CSF-Aβ_42_ levels with relatively lower PET uptake values than expected, and as a result worse cognitive functioning.

We show that the imbalance model generalized, with a similar range in Aβ-aggregation and Aβ-severity scores, to an independent and early cohort with different CSF kits and PET tracers, highlighting the applicability beyond ADNI. However, it should be noted that while similar trends were observed, not all associations with Aβ-aggregation reached statistical significance in the validation cohort. This is not unexpected, considering the smaller sample size and early nature of this cohort, resulting in limited statistical power. In addition, this finding is in line with the observation that in the discovery cohort associations with CSF p-tau and cognition were driven by CI individuals. Hence, although it goes beyond the scope of this study, it is of interest for future research to investigate whether findings can be replicated in a more similar cohort to ADNI.

Taken all results together, our findings provide additional evidence to the hypothesis that an imbalance between soluble and aggregated Aβ might reflect distinct disease pathways toward full Aβ biomarker abnormality, with an imbalance toward more soluble Aβ being related to worse outcomes. Although these effects were exacerbated at more advanced levels of Aβ pathology, they were already present in an early population. Thus, Aβ biomarker imbalance might allow for the identification of individuals at risk of clinical progression, which could be relevant for selection strategies in clinical trials. In line, it was recently shown that anti-amyloid drugs targeting soluble Aβ might be more successful in slowing cognitive decline.^50^ We show that relatively more soluble Aβ occurs not only in early disease stages, but across the AD continuum, potentially identifying patients who would benefit most from current treatments.

Strengths of this study include the use of a discovery and validation cohort, illustrating the applicability of the continuous imbalance model across heterogeneous study populations, PET tracers, and CSF kits. This study also has several limitations. First, although we tested several methodological factors, several preanalytical and postanalytical factors such as handling of CSF samples or off-target PET ligand binding could have contributed to Aβ biomarker imbalance. However, in the current work, we attempted to minimize the effect of these methodological factors by harmonizing and standardizing outcome measures across multiple cohorts and study sites. Second, the CSF/PET imbalance measure is strongly dependent on model fit, and as a result, large samples are needed to accurately assess deviations or an imbalance in the relationship between CSF and PET biomarkers. This could potentially be of concern for other study cohorts with relatively fewer data points, leading to less accurate estimations of imbalance. However, in our validation cohort, which was substantially smaller than the discovery cohort, model fit and ranges in Aβ-aggregation scores comparable with the discovery cohort were observed.

In conclusion, we showed a more general framework of Aβ biomarker imbalance permeating the full AD continuum. We observed that such continuous measure of Aβ biomarker imbalance was associated with genetic risk, AD-associated CSF biomarkers, and cognition. These findings suggest that Aβ biomarker imbalance might represent more than methodological error, possibly reflecting disease heterogeneity of potential value to clinical trials.

## Appendix 1 Authors

**Table:**

Name	Location	Contribution
Sophie E. Mastenbroek, MSc	Department of Radiology and Nuclear Medicine, Vrije Universiteit Amsterdam, Amsterdam University Medical Center, location VUmc; Amsterdam Neuroscience, Brain Imaging, the Netherlands; Clinical Memory Research Unit, Department of Clinical Sciences Malmö, Lund University, Sweden	Drafting/revision of the manuscript for content, including medical writing for content; major role in the acquisition of data; study concept or design; analysis or interpretation of data
Arianna Sala, PhD	Division of Clinical Geriatrics, Center for Alzheimer Research, Department of Neurobiology, Care Sciences and Society, Karolinska Institutet, Stockholm, Sweden; Coma Science Group, GIGA-Consciousness, University of Liège; Centre du Cerveau2, University Hospital of Liège, Belgium	Drafting/revision of the manuscript for content, including medical writing for content; study concept or design; analysis or interpretation of data
David Vállez García, PhD	Department of Radiology and Nuclear Medicine, Vrije Universiteit Amsterdam, Amsterdam University Medical Center, location VUmc; Amsterdam Neuroscience, Brain Imaging, the Netherlands	Drafting/revision of the manuscript for content, including medical writing for content; major role in the acquisition of data
Mahnaz Shekari, PhD	Barcelonaβeta Brain Research Center (BBRC), Pasqual Maragall Foundation; IMIM (Hospital del Mar Medical Research Institute), Barcelona; Centro de Investigación Biomédica en Red de Fragilidad y Envejecimiento Saludable, Instituto de Salud Carlos III, Madrid; Universitat Pompeu Fabra, Barcelona, Spain	Drafting/revision of the manuscript for content, including medical writing for content; major role in the acquisition of data
Gemma Salvadó, PhD	Clinical Memory Research Unit, Department of Clinical Sciences Malmö, Lund University, Sweden; Barcelonaβeta Brain Research Center (BBRC), Pasqual Maragall Foundation, Spain	Drafting/revision of the manuscript for content, including medical writing for content
Luigi Lorenzini, PhD	Department of Radiology and Nuclear Medicine, Vrije Universiteit Amsterdam, Amsterdam University Medical Center, location VUmc; Amsterdam Neuroscience, Brain Imaging, the Netherlands	Drafting/revision of the manuscript for content, including medical writing for content; major role in the acquisition of data
Leonard Pieperhoff, MSc	Department of Radiology and Nuclear Medicine, Vrije Universiteit Amsterdam, Amsterdam University Medical Center, location VUmc; Amsterdam Neuroscience, Brain Imaging, the Netherlands	Drafting/revision of the manuscript for content, including medical writing for content; major role in the acquisition of data
Alle Meije Wink, PhD	Department of Radiology and Nuclear Medicine, Vrije Universiteit Amsterdam, Amsterdam University Medical Center, location VUmc; Amsterdam Neuroscience, Brain Imaging, the Netherlands	Drafting/revision of the manuscript for content, including medical writing for content; major role in the acquisition of data
Isadora Lopes Alves, PhD	Brain Research Center, Amsterdam, the Netherlands	Drafting/revision of the manuscript for content, including medical writing for content; major role in the acquisition of data
Robin Wolz, PhD	IXICO, London, United Kingdom	Drafting/revision of the manuscript for content, including medical writing for content; major role in the acquisition of data
Craig Ritchie, PhD	Centre for Clinical Brain Sciences, University of Edinburgh, Scotland, United Kingdom	Drafting/revision of the manuscript for content, including medical writing for content; major role in the acquisition of data
Mercè Boada, MD, PhD	Ace Alzheimer Center Barcelona, Universitat Internacional de Catalunya; Networking Research Center on Neurodegenerative Diseases (CIBERNED), Instituto de Salud Carlos III, Madrid, Spain	Drafting/revision of the manuscript for content, including medical writing for content; major role in the acquisition of data
Pieter Jelle Visser, MD, PhD	Alzheimer Center Amsterdam, Neurology, Vrije Universiteit Amsterdam, Amsterdam UMC location VUmc; Amsterdam Neuroscience, Neurodegeneration; Alzheimer Center Limburg, School for Mental Health and Neuroscience, Maastricht University, the Netherlands; Division of Neurogeriatrics, Department of Neurobiology, Care Sciences and Society, Karolinska Institutet, Stockholm, Sweden	Drafting/revision of the manuscript for content, including medical writing for content; major role in the acquisition of data
Marco Bucci, PhD	Division of Clinical Geriatrics, Center for Alzheimer Research, Department of Neurobiology, Care Sciences and Society, Karolinska Institutet; Theme Inflammation and Aging, Karolinska University Hospital, Stockholm, Sweden	Drafting/revision of the manuscript for content, including medical writing for content
Gill Farrar, PhD	GE Healthcare, Amersham, United Kingdom	Drafting/revision of the manuscript for content, including medical writing for content
Oskar Hansson, MD, PhD	Clinical Memory Research Unit, Department of Clinical Sciences Malmö, Lund University; Memory Clinic, Skåne University Hospital, Malmö, Sweden	Drafting/revision of the manuscript for content, including medical writing for content
Agneta K. Nordberg, MD, PhD	Division of Clinical Geriatrics, Center for Alzheimer Research, Department of Neurobiology, Care Sciences and Society, Karolinska Institutet; Theme Inflammation and Aging, Karolinska University Hospital, Stockholm, Sweden	Drafting/revision of the manuscript for content, including medical writing for content
Rik Ossenkoppele, PhD	Clinical Memory Research Unit, Department of Clinical Sciences Malmö, Lund University, Sweden; Alzheimer Center Amsterdam, Neurology, Vrije Universiteit Amsterdam, Amsterdam UMC location VUmc, the Netherlands	Drafting/revision of the manuscript for content, including medical writing for content; study concept or design; analysis or interpretation of data
Frederik Barkhof, MD, PhD	Department of Radiology and Nuclear Medicine, Vrije Universiteit Amsterdam, Amsterdam University Medical Center, location VUmc; Amsterdam Neuroscience, Brain Imaging, the Netherlands; Centre for Medical Image Computing, and Queen Square Institute of Neurology, UCL, London, United Kingdom	Drafting/revision of the manuscript for content, including medical writing for content; major role in the acquisition of data; analysis or interpretation of data
Juan Domingo Gispert, PhD	Barcelonaβeta Brain Research Center (BBRC), Pasqual Maragall Foundation; IMIM (Hospital del Mar Medical Research Institute), Barcelona; Centro de Investigación Biomédica en Red de Fragilidad y Envejecimiento Saludable, Instituto de Salud Carlos III, Madrid, Spain	Drafting/revision of the manuscript for content, including medical writing for content; major role in the acquisition of data; study concept or design; analysis or interpretation of data
Elena Rodriguez-Vieitez, PhD	Division of Clinical Geriatrics, Center for Alzheimer Research, Department of Neurobiology, Care Sciences and Society, Karolinska Institutet, Stockholm, Sweden	Drafting/revision of the manuscript for content, including medical writing for content; study concept or design; analysis or interpretation of data
Lyduine E. Collij, PhD	Department of Radiology and Nuclear Medicine, Vrije Universiteit Amsterdam, Amsterdam University Medical Center, location VUmc; Amsterdam Neuroscience, Brain Imaging, the Netherlands; Clinical Memory Research Unit, Department of Clinical Sciences Malmö, Lund University, Sweden	Drafting/revision of the manuscript for content, including medical writing for content; major role in the acquisition of data; study concept or design; analysis or interpretation of data

## Appendix 2 Coinvestigators

**Table:**

Coinvestigators are listed at Neurology.org.

## Glossary

Aβ: β-amyloid
AD: Alzheimer disease
ADNI: Alzheimer's Disease Neuroimaging Initiative
ADAS-Cog: Alzheimer's Disease Assessment Scale-Cognitive Subscale
ADNI-EF: Alzheimer's Disease Neuroimaging Initiative executive functioning
ADNI-MEM: Alzheimer's Disease Neuroimaging Initiative memory
ALFA+: Alzheimer's and Family cohort
AMYPAD: Amyloid Imaging to Prevent Alzheimer's Disease
CDR: Clinical Dementia Rating
CI: cognitively impaired
CU: cognitively unimpaired
CL: Centiloid
EPAD-LCS: European Prevention of Alzheimer's Dementia Longitudinal Cohort Study
FACEHBI: Fundació ACE Healthy Brain Initiative
FBB: [18F]Florbetaben
FBP: [18F]florbetapir
FLAIR: fluid-attenuated inversion recovery
FMM: [18F]Flutemetamol
IQR: interquartile range
LEAP: learning embeddings for atlas propagation
MBq: megabecquerel
MCI: mild cognitive impairment
MMSE: Mini-Mental State Examination
PC: parent cohort
p.i.: postinjection
PNHS: Prognostic and Natural History Study
p-tau: phosphorylated-tau
SUVr: standardized uptake value ratio
t-tau: total-tau
WMH: white matter hyperintensity
